# Comparison of the Performance of Two Galactomannan Detection Tests: Platelia Aspergillus Ag and Aspergillus Galactomannan Ag Virclia Monotest

**DOI:** 10.1128/spectrum.02626-21

**Published:** 2022-03-09

**Authors:** Alba Leyva Calero, Roberto Alonso, Ignacio Gadea, María Dolores Montero Vega, Marta Martín García, Patricia Muñoz, Marina Machado, Emilio Bouza, Julio García-Rodríguez

**Affiliations:** a Department of Biochemistry, Immunology and Molecular Parasitology, Universidad de Granada, Granada, Spain; b Department of Clinical Microbiology and Infectious Diseases, Hospital General Universitario Gregorio Marañón, Madrid, Spain; c Instituto de Investigación Biosanitaria Gregorio Marañón, Madrid, Spain; d CIBER de Enfermedades Respiratorias CIBERES, Barcelona, Spain; e Department of Medicine, Facultad de Medicina, Universidad Complutense, Madrid, Spain; f Department of Clinical Microbiology, Fundación Jimenez Díaz, Autonomous University, Madrid, Spain; g Department of Clinical Microbiology, Hospital La Paz, Autonomous University, Madrid, Spain; Geisel School of Medicine at Dartmouth

**Keywords:** galactomannan, Platelia, VirClia, invasive aspergillosis, *Aspergillus*, diagnostic test, chemiluminescence, enzyme-linked immunosorbent assay

## Abstract

The use of nonculture-based biomarkers such as the determination of galactomannan is sought for the diagnosis of invasive aspergillosis. To investigate the comparative yield of two tests for the detection of galactomannan in patients with or without proven or probable invasive aspergillosis. Overall, 327 samples (327 patients) were analyzed in a retrospective/prospective study performed in 3 hospitals in Madrid, comparing the determination results in serum or bronchoalveolar lavage of two techniques for galactomannan detection, namely, Platelia Aspergillus Ag (Bio-Rad) and Aspergillus galactomannan Ag Virclia Monotest (Vircell S.L.), following the manufacturer’s instructions. Both techniques can automate the process, but the second technique has the advantage of individual processing and assembly of each sample without the need for the additional expense of single-dose strips in controls. In total, 288 of the 327 tests performed showed concordant results between both techniques. The agreement between both methods was к = 0.722, and the correlation between indices was ρ = 0.718. Only 39 samples showed discordant results. In those 39 cases, there were 15 patients with proven or probable invasive aspergillosis criteria. For the samples with clinical criteria as a reference, the areas under the curve of the receiver operating characteristic (ROC) curve were 0.962 for Platelia and 0.968 for VirClia. The VirClia test has been proven to be an alternative for diagnosis due to its friendlier automated format than that of the usual Platelia routine test. The VirClia test also allows individual action and, therefore, a more immediate clinical response.

**IMPORTANCE** Invasive mycoses are increasingly present in immunosuppressed or hospitalized patients with serious illnesses, leading to high rates of morbidity and mortality. Invasive aspergillosis is an infection caused, in a percentage greater than 50%, by the genus Aspergillus. It is vitally important to make an early diagnosis that leads to the application of antifungals in the initial stage of the infection. Therefore, tools are required to help with the early diagnosis of the infection. This comparative study of two enzyme immunoassays is based on the detection of galactomannan antigen in serum and bronchoalveolar lavage samples. A new design based on chemiluminescence and presented in an automated single-dose format is compared to a conventional ELISA technique marketed for years. The results obtained from the prospective and retrospective study indicate a high correlation and degree of agreement between both techniques, as well as in their diagnostic performance.

## INTRODUCTION

Invasive fungal infection (IFI) is becoming increasingly important due to a series of circumstances, including the growth of an elderly and immunosuppressed population with multiple comorbidities in which the control of bacterial infections is more effective (1–4).

Invasive aspergillosis (IA) is the second most common IFI, behind invasive candidiasis (IC); at present, the population at risk is composed not only of neutropenic patients but also of a growing group of cases with other comorbidities, including SARS-CoV-2 infection (1–3). The signs and symptoms of IA are often nonspecific. It is challenging to distinguish colonization from infection; blood cultures are practically always negative, and it is frequently difficult or impossible to perform invasive techniques in order to obtain proper samples. In these circumstances, the use of nonculture-based biomarkers is therefore essential (5). IA biomarkers such as galactomannan (GLM) or 1–3 β-D glucan (BDG) are useful in clinical practice (6).

Galactomannans are cell wall components of some fungi such as Aspergillus and are released during tissue growth. The detection of galactomannan in blood or bronchoalveolar lavage (BAL) is useful for diagnosing IA in humans. The reference technique for the detection of GLM has been the Platelia Aspergillus (Bio-Rad) test, but in its current format, marketed as a sandwich enzyme-linked immunosorbent assay (ELISA) microtiter plate, this technique makes it very difficult to be used with single samples, and it is not able to provide a “same-day result” in many institutions (7–9).

A new single test for GLM, Aspergillus galactomannan Ag VIRCLIA MONOTEST, which uses chemiluminescence detection and runs in an automated analyzer developed by Vircell, could, in the absence of a clinical evaluation, respond to these new diagnostic needs.

Our study consists of comparing the performance of two procedures to determine GLM in serum or BAL fluid: Aspergillus galactomannan Ag VIRCLIA MONOTEST (Vircell S.L.) versus Platelia Aspergillus Ag (Bio-Rad).

## RESULTS

During the study period, a first GLM determination was requested from 327 patients; 120 were obtained retrospectively (25 from healthy individuals who were considered negative controls and 95 from patients with proven or probable IA criteria), and the remaining 207 were prospectively obtained from daily GLM applications.

Globally, 106 determinations were positive for one technique or another; 80 of them were positive with Platelia (75.5%), and 101 were positive with VirClia (95.3%). Of the positive samples, 75 were concordant in both techniques, 5 were discordant in Platelia and 26 were discordant in VirClia (Table 1).

**TABLE 1:** Comparison between both techniques

Classification	Platelia positive	Platelia negative	Overall
VirClia positive	75	26	101
VirClia doubtful	3	8	11
VirClia negative	2	213	215
Overall	80	247	327

Overall, 249 determinations were performed in serum samples and 78 in BAL fluid samples (Table 2). The results were concordant (Table 1) in 291 out of 327 samples (89.0%) if we counted doubtful samples as positive and in 297 out of 327 samples (93.7%) if we counted doubtful samples as negative.

**TABLE 2:** Type of samples evaluated

Centers	Overall	Serum	BAL fluid
FJD	113	53	60
GMH	120	108	12
LA PAZ H	94	88	6
Overall	327	249	78

The degree of agreement between both methods was analyzed by Cohen’s kappa coefficient (к), showing a value of 0.722 (standard error = 0.042; 95% confidence interval [CI] = 0.639 to 0.804), that is, a “substantial” agreement between evaluators. The correlation between index values was analyzed using Spearman´s correlation coefficient (ρ), and the value obtained was ρ = 0.718 (P: 0.000; 95% CI = 0.662 to 0.767).

**FIG 1:**
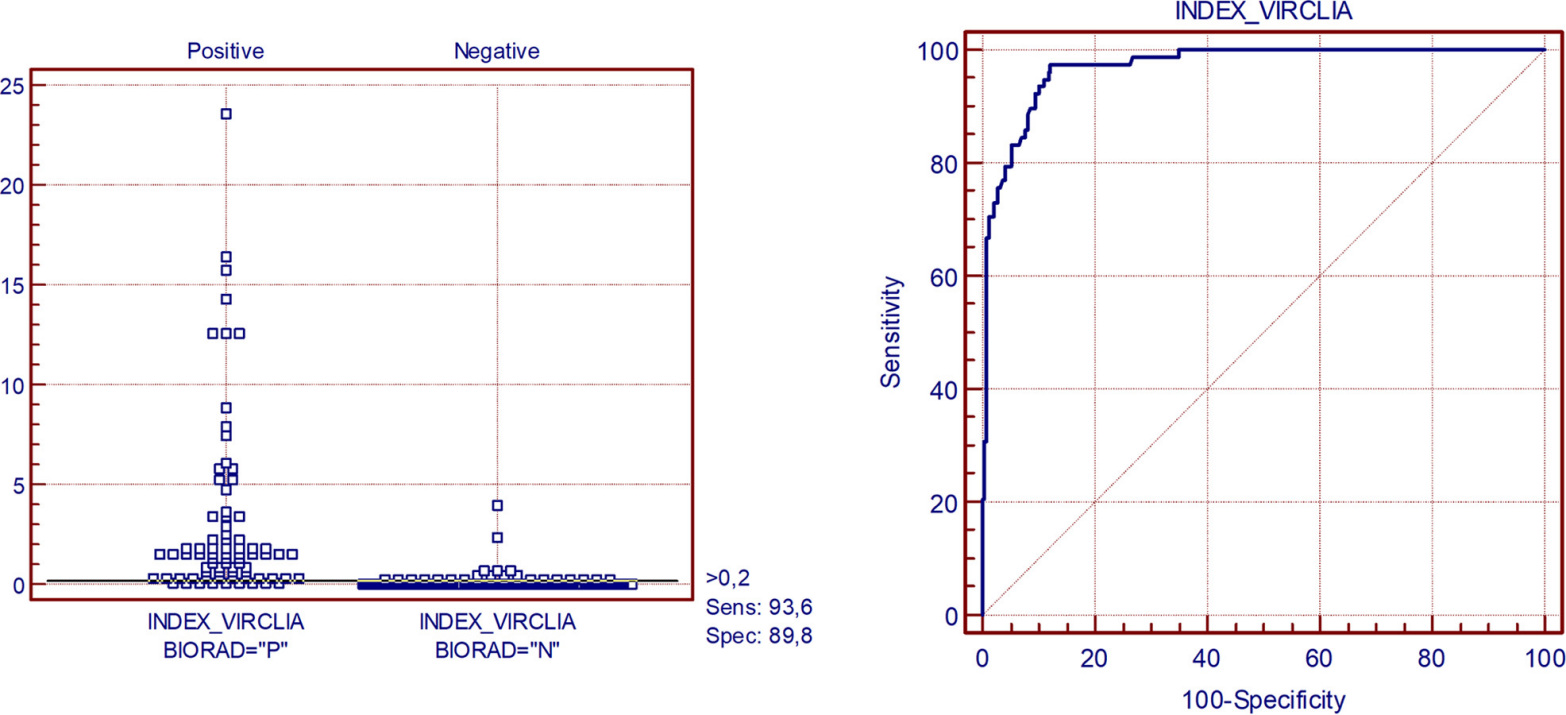
ROC Curve. VirClia kit using Platelia kit as a reference. ROC curve sample size of positive group N = 80 and sample size of negative group N = 247. Area under the ROC curve was 0.97 (95% CI = 0.946 to 0.986).

Depending on the discrimination threshold values, we obtained a varied proportion of true positives versus that of false positives through an ROC curve (Fig. 1). The new VirClia technique was compared using Platelia as a reference kit; the optimal point was established at VirClia INDEX = 0.2, obtaining a sensitivity of 93.8% and a specificity of 89.5%.

With respect to the retrospective study, overall, 18 of 39 discordant samples were resolved according to the clinical classification: 15 samples were negative for Platelia and positive for VirClia, and 3 samples were positive for Platelia and negative for VirClia. In all cases, the patients had criteria for proven or probable IA. Against this clinical gold standard, the Platelia/VirClia results were compared as follows (Table 3): the sensitivity, specificity, positive predictive value (PPV) and negative predictive value (NPV) were 63.2%, 100%, 100%, and 41.7%, for Platelia, respectively, and 80.9%, 100%, 100%, and 59.5% for VirClia.

**TABLE 3:** Results according to clinical classification^*a*^

	Platelia	VirClia
Result correlation	N	N
True positive	60	72
False positive	0	0
True negative	25	25
False negative	35	17
Borderline	0	6
Total	120	120
Sensitivity (%)	63,2	80,9
Specificity (%)	100	100
PPV	100	100
NPV	41.7	59.5

a Results obtained with the EORTC/MSG criteria as gold standard.

**FIG 2:**
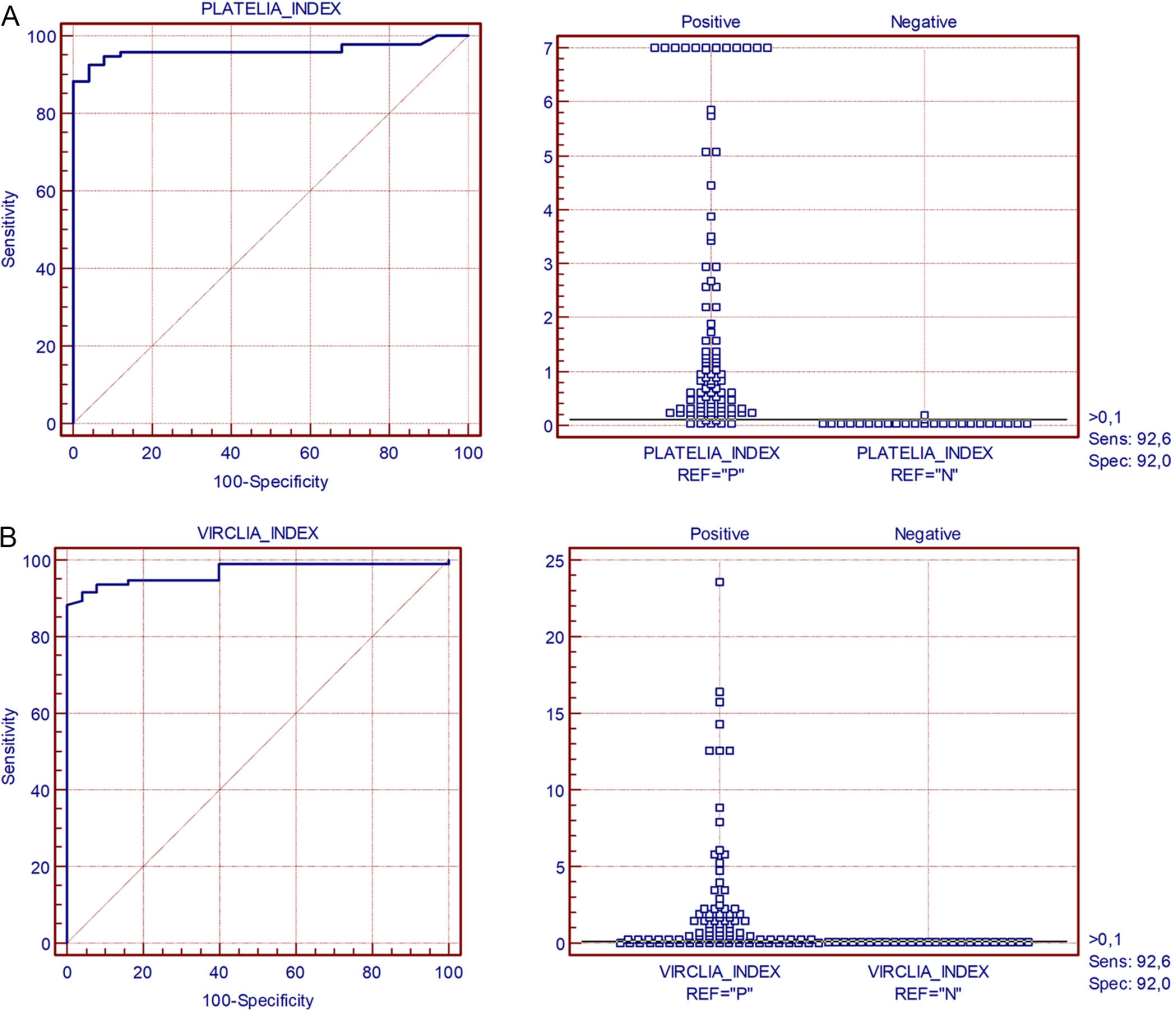
ROC Curve. VirClia and Platelia kit using EORTC/MSG criteria as reference. A) Platelia ROC curve: sample size of positive group N = 95 and sample size of negative group N = 25. Area under the ROC curve was 0.962 (95% CI = 0.910 to 0.988). B) VirClia ROC curve: sample size of positive group N = 95 and sample size of negative group N = 25. Area under the ROC curve was 0.968 (95% CI = 0.919 to 0.991).

Following the retrospective study, using the clinical criterion of proven or probable according to EORT/MSG as a reference, values of the areas under the curve of the ROC curve were obtained, with 0.962 for the Platelia kit and 0.968 for the VirClia kit (Fig. 2).

**FIG 3:**
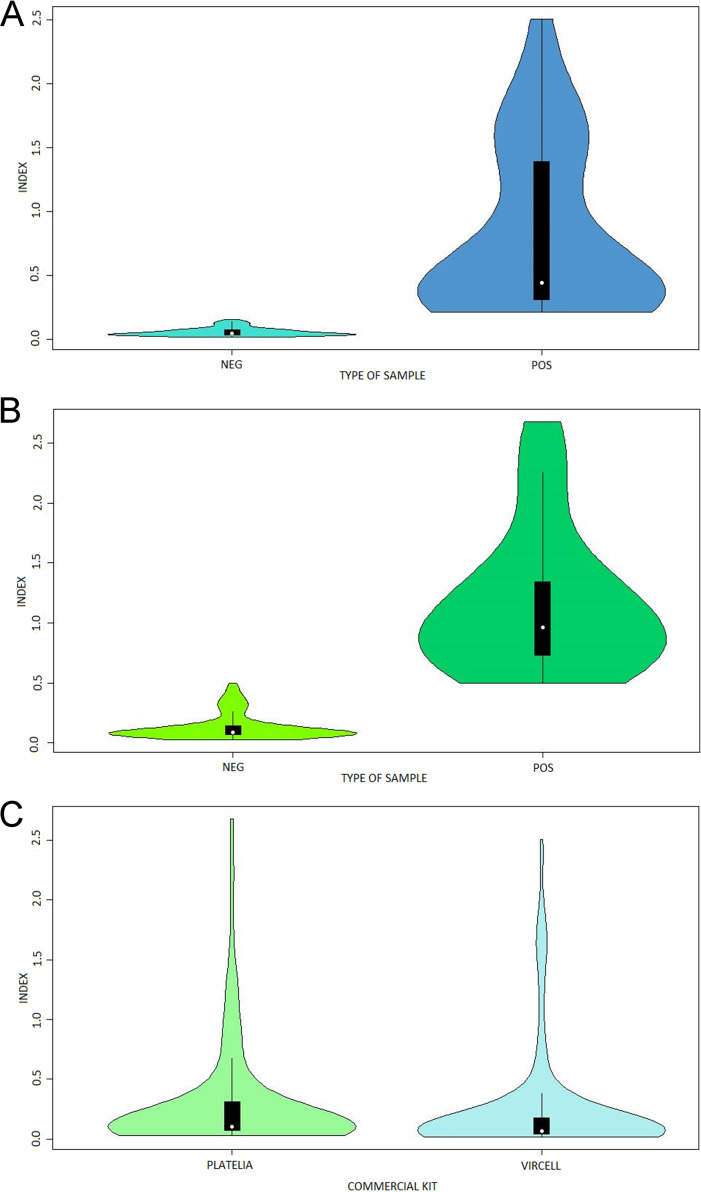
Vilion plots. Data distribution and probability density for negative and positive samples individually analyzed for Aspergillus galactomannan Ag VIRCLIA MONOTEST (Fig. 3A) and Platelia Aspergillus Ag (Fig. 3B). Data distribution and probability density for negative and positive samples analuzed as whole for both techniques (Fig. 3C). The criteria for classifying samples have been recommended by the manufacturer (negative sample index <16 and positive sample index ≥2 for VirClia; and negative sample index <0.5 and positive sample index ≥0.5 for Platelia). The borderline samples have not been consider for this analysis.

The distribution of the global data and the probability density of the two methods were analyzed using violin plots. The distribution and density of negative and positive samples were analyzed individually in the VirClia and Platelia kits (Fig. 3A, Fig. 3B). Moreover, the global set of index results for each method was analyzed (Fig. 3C).

## DISCUSSION

Our study shows that the determination of GLM by the Vircell procedure provides results comparable or superior to those of the Bio-Rad system, with the advantage that the VirClia test can be performed for a single sample in a short time.

The diagnosis of IA remains a severe clinical problem for many reasons. First, the predisposing diseases and risk factors vary, including many nonneutropenic patients with nonhematologic underlying conditions (10–12).

In addition, culture-based procedures are of relatively low benefit and often have ambiguous interpretation (6, 13). Furthermore, histological confirmation is limited by the difficulty of obtaining tissue samples, and PCR techniques have important limitations (1, 3, 14–16). This explains the need to use biomarkers such as 1–3 β-D glucan or GLM in blood or BAL fluid samples (6, 10, 17–25).

The Platelia Aspergillus Ag kit (Bio-Rad) was approved by the FDA in 2003 and detects the presence of GLM produced by Aspergillus during its invasive growth. The use of this test has been particularly beneficial in neutropenic patients and has been limited by the existence of false positives in patients exposed to products that may have been contaminated by fungal remains during manufacture; examples include certain antibiotics and even some bags used for collection and administration of platelets (26, 27). Occasionally, patients with well-demonstrated bacterial diseases show positive GLM results, but it is often difficult to rule out the presence of coinfections in some of those cases detected by the tests (28–33). It is also known that intravenous immunoglobulin (IVIG) administration may produce false-positive GLM ELISA results (34).

The latest reviews and meta-analyses have confirmed that the detection of GLM in BAL fluid is more sensitive than in serum, both in hematological and nonhematological patients (13, 35, 36). Other situations in which the determination of GLM may be useful are in extrapulmonary forms of IA, such as the case of cerebrospinal fluid for cerebral localizations (37) or peritoneal fluid for the diagnosis of peritonitis (38). Recently, GLM has also been considered useful in the follow-up of high-risk patients in hospitals undergoing renovations (39), as a prognostic marker of chronic aspergillosis, and as a diagnostic test for disseminated fusariosis (39, 40).

A meta-analysis of high-risk patients combining GLM and PCR weekly determination included 13 studies and 1,670 patients (41). The study concluded that the negativity of all the tests makes it possible to obviate the need to administer antifungal agents, with an NPV of 100%, while the presence of at least 2 positive tests is highly suggestive of active infection, with a PPV of 88%.

The Platelia GLM test has been the international reference for many years. However, the format currently marketed with a sandwich ELISA microtiter plate makes it very difficult to use with only one or a few samples and requires sample accumulation and batching. Initially, a latex agglutination test to detect GLM antigen existed in a commercialized form, which was substituted by sandwich ELISA due to the greater analytical sensitivity of the latter. The ELISA method is useful when systematically studying a large number of patients at risk of IA. New formats are now needed to facilitate its use with a single sample. The new single test using chemiluminescence developed by Vircell is a very promising tool and, according to our study, may be a substitute for the Platelia Aspergillus Ag kit produced by Bio-Rad due to high correlation (ρ = 0.718) and agreement (к = 0.722) between both techniques and their diagnostic performance comparable with the AUC under ROC curves of 0.962 and 0.968 for Platelia and VirClia, respectively. The VirClia kit has the advantage of including the controls in the single-dose strip, so the expense of control strips is unnecessary, and the time required to obtain results is less than an hour. This assay also is more affordable than the Platelia kit. In our study, we calculated the hypothetical delay in response times in terms of providing results to patients if using GLM batch testing; the times were up to 48 h for Platelia and up to 2.5 h for VirClia depending on the availability of the autoanalyzer (42–46).

VirClia guarantees processing of the sample the moment that it is received, in addition to exhibiting objectivity in the reading and sensitivity provided by the immunoenzymatic method compared to products based on the lateral flow technique recently developed and marketed (47, 48).

Our study is limited by the sample size and the lack of a clear-cut gold standard to compare diagnostic tests for IA. However, our methodology allows us to compare the results of the VirClia test with those obtained with the Platelia test.

In our opinion, the VirClia test can replace the Platelia test whenever an individual use of the test is required, a quicker response is clinically necessary, or a policy of saving limited resources is implemented. The diagnosis strategy for the future will probably involve the combination of fungal biomarkers. The screening of IA is also essential to decrease the empirical use of antifungals. Further prospective and comparative studies are needed to establish the performance of the VirClia test.

## MATERIALS AND METHODS

From January 2019 to November 2019, three tertiary hospitals in the city of Madrid (Spain) participated in the study: Gregorio Marañón University Hospital (GMH), La Paz University Hospital (La Paz H) and Clínica de la Concepción (Fundación Jiménez Díaz, FJD). One of the centers (GMH) mainly provided retrospective samples from patients with known proven or probable IA, stored at −80°C, to obtain a larger number of positive samples. In the other two hospitals, samples were included prospectively upon clinical request for GLM determination. Only samples from adult patients were included, and a single sample per patient was evaluated (the first sample). All samples were tested by the employees of each facility.

In all samples, Platelia Aspergillus Ag (Bio-Rad) and Aspergillus galactomannan Ag VIRCLIA MONOTEST (Vircell S.L.) were run in parallel and according to the manufacturer’s recommendations. In both cases, samples were pretreated with EDTA and heated to dissociate immune complexes and precipitate proteins that could interfere with the test. The process consisted of heating the sample with the EDTA solution for approximately 6 min in a heat block at 120°C and then centrifuging for 10 min at 10000 × <ig>. The supernatant was tested using both techniques.

ELISA is an immunoassay technique where antigen-antibody interactions exist and the final product is detectable with spectrophotometry. Chemiluminescent immunoassay (CLIA) is also an immunoassay technique, but the final product is light. The CLIA technique exhibits better sensitivity than the chromogenic technique.

Testing with the Platelia Aspergillus Ag kit (Bio-Rad) was performed on serum and BAL fluid samples in an EVOLIS TWIN autoanalyzer (Bio-Rad). Briefly, microwell plates coated with monoclonal anti-GLM rat antibody were incubated with pretreated clinical samples and conjugate reagent (peroxidase-linked monoclonal antibody). After a washing step, 3,3′,5,5′-tetramethylbenzidine (TMB) chromogen was added, followed by further incubation. The reaction was stopped by adding an acid solution, and the absorbance was read at 450 nm. The threshold for positivity was an index set at 0.5 as proposed by Bio-Rad. A cutoff control was included in the kit and used to calculate an index for each sample (Index=OD Sample/OD cutoff). The duration of the technique per patient was approximately 2 h and entailed the use of 4 wells of the plate for the controls each time the assay was set up.

Testing with the Aspergillus galactomannan Ag VIRCLIA MONOTEST was performed on a VirClia autoanalyzer. Although the reaction was equivalent to that with the Bio-Rad kit, the main differences were the use of single strips, which included all necessary reagents (including negative control and calibrator) for each sample, and the use of a chemiluminescent substrate (luminol) instead of a colorimetric substrate. Similar to Bio-Rad, the rat anti-GLM monoclonal antibody-coated microwell plates were incubated with pretreated clinical specimens, and after the washing step, the plate was incubated with a conjugate reagent (peroxidase-linked monoclonal antibody). Then, after the second washing step, the chemiluminescent substrate was added, followed by further incubation. A calibrator control was included in the kit and used to calculate an index for each sample (Index= Relative Light Units (RLU) Sample/Relative Light Units (RLU) calibrator). The considered preliminary cutoff for positivity, as proposed by Vircell, was 0.2. The time required for diagnosis of a patient was slightly more than an hour.

The theoretical time to provide results if samples were received regularly was calculated.

The results of the comparison between both techniques were classified as follows:

Agreement: Both techniques have a positive or negative result.

Disagreement: One of the techniques gives a positive result and the other a negative result.

The discordant results, in the retrospective study, were compared with a clinical reference pattern, consisting of establishing the presence of clinical criteria of proven or probable IA as the “gold standard” following the criteria of the European Organization for Research and Treatment of Cancer and the Mycoses Study Group Education and Research Consortium (EORTC/MSG) (49) at the time of sampling.

### Statistical analysis.

The results between methods were compared through Cohen’s kappa coefficient (к) and Spearman´s correlation coefficient (ρ). Through a receiver operating characteristic (ROC) curve, we obtained a proportion of true positives versus that of false positives for the global study comparing the VirClia and Platelia kits as well as for a retrospective study using the EORTC/MSG criteria as a reference. The results were compared to define agreement and disagreement rates between techniques using the statistical programs Medcalc (version 5.00.017) and RStudio (version 3.5.3; “xlsx” version 0.6.3; “ggplot2” version 3.3; “plyr” version 1.8.6; “reshape2” version 1.4.4). False-positive and false-negative results from the retrospective study were further analyzed considering clinical information.

### Ethics approval.

This study was approved by the institutional ethics committee (Comité Ético de Investigación Clínica del Hospital Gregorio Marañón [CEIC-A1]). The need for informed consent was waived due to the noninterventional design of the study.
